# The use of brain stimulation in the rehabilitation of walking disability in patients with multiple sclerosis: A randomized double-blind clinical trial study

**Published:** 2019-04-04

**Authors:** Shahram Oveisgharan, Zahra Karimi, Siamak Abdi, Hajir Sikaroodi

**Affiliations:** 1 Iranian Center of Neurological Research, Neuroscience Institute, Tehran University of Medical Sciences, Tehran, Iran; 2 Department of Neurology, Shariati Hospital, Tehran University of Medical Sciences, Tehran, Iran

**Keywords:** Multiple Sclerosis, Transcranial Direct Current Stimulation, Mobility Limitation

## Abstract

**Background:** Transcranial direct current stimulation (tDCS) of the primary motor cortex of the lower limb has been exploited in the treatment of patients with stroke and spastic lower limb paresis. We examined this stimulation efficacy in the treatment of multiple sclerosis (MS)-related walking disability.

**Methods:** In a single-center randomized double-blind clinical trial study, 13 patients with MS and walking disability and Expanded Disability Status Scale (EDSS) score of 3 to 6 were randomized to the real and sham stimulation groups. In the real tDCS stimulation, 7 patients received anodal 2.5 mA stimulation at 1 cm anterior to the C_z_ point for 30-minute daily sessions in 7 consecutive days. The other group received sham stimulation with the same protocol. The primary outcome of the trial was change in the Timed 25-Foot Walk (T25-FW) from before to after the stimulation. We also assessed the Multiple Sclerosis Walking Scale-12 (MSWS-12). We employed linear mixed effects model to examine the efficacy of tDCS stimulation on changing the outcomes.

**Results:** On average, patients who received real tDCS stimulation walked faster after 7 sessions of stimulation [Estimate = -2.7, standard error (SE) = 1.3, P = 0.049], while walking speed of sham stimulation recipients did not change. For every session of stimulation, recipients of real tDCS stimulation spent 2.7 seconds less for walking the 25 feet. Real tDCS stimulation was not effective in improving MSWS-12 scores.

**Conclusion:** tDCS stimulation of the lower limb motor cortex speeded up patients with MS in walking, but without improvement in patients’ mobility in daily activities.

## Introduction

Walking disability is a common multiple sclerosis (MS) health problem affecting many patients with MS when several years have passed since disease onset. In a longitudinal study of Swedish patients with MS, more than half of the moderate to severe patients had walking disability after 10 years of follow up.^1^ Following 806 patients with relapsing remitting MS (RRMS) for at least 16 years, investigators found a median of 18 years as the time to walking disability since disease onset.^2^ Moreover, walking disability is the greatest concern for patients with MS,^3^ and impaired mobility is associated with reduction in quality of life, activities of daily living, and productivity reported by patients with MS.^4^ So, looking for therapies targeting walking disability of patients with MS is a clinical priority. 

The main reason for walking disability of patients with MS is increased lower limb spasticity. It is believed that inadequate plasticity in the brains of patients with MS causes MS symptoms, including spasticity, to appear and to progress.^5^ Long-term potentiation (LTP) and long-term depression (LTP) are the major mechanisms in the plasticity phenomenon,^6^ and both animal and human studies have shown increased LTP in MS,^5^ possibly as a compensatory mechanism to neuronal damage and synaptic loss. Transcranial magnetic stimulation, as a non-invasive way to induce LTP, has been used in patients with MS to improve their lower limb spasticity.^7^^,^^8^

Transcranial direct current stimulation (tDCS) is another noninvasive method to modulate synaptic plasticity most possibly through enhancing the LTP and LTD effects.^9^ Investigators have successfully used this technique to improve complaints of patients with MS such as chronic pain,^10^ tactile sensory deficits,^11^ and fatigue.^12^ Moreover, studies on patients with stroke and limb paresis have showed the effectiveness of tDCS in improving stroke-related paresis^13^ and walking disability.^14^ Therefore, we conducted this randomized clinical trial study to examine the efficacy of tDCS in improving walking ability of patients with MS.

## Materials and Methods

Patients with MS who were under treatment at the MS clinic of Shariati Hospital in Tehran, Iran, were invited to participate. Moreover, by joining MS groups at Telegram Messenger (Telegram Messenger LLP, London, UK), we invited their friends diagnosed with MS. At study enrollment, we clinically evaluated patients to verify their MS diagnosis according to McDonald revised criteria.^15^ We recruited patients with MS who complained of difficulty in walking due to MS, had spastic paraparesia in the clinical examination, and did not have any clinical or radiological disease activity during the last 2 months. Patients were excluded if their Expanded Disability Status Scale (EDSS) score was more than 6, had metals in the skull or the brain, were younger than 18 years old, or were consumers of antispastic medications (such as baclofen). Patients signed the consent forms, and the study was approved by the Ethics Committee of the Tehran University of Medical Sciences, Iran. 

Using the SPSS random number generators package, we randomized our recruited patients into the intervention and sham groups based on the real vs. sham tDCS stimulation they received. We programmed block random allocation with no stratum following the Arifin’s paper.^16^

The current, generated by DC stimulators made in Iran, was delivered through saline-soaked sponges 4 × 4 cm^2^ in size. The real stimulation was 2.5 mA for 30 minutes with 10 seconds fade-in and fade-out, while the sham stimulation was 2.5 mA lasted for 30 seconds with the same fade-in and fade-out duration. 

In both real and sham groups, the sponge under the anode was centered at 1 cm anterior to the C_z_ in the 10/20 electroencephalogram (EEG) system, and the cathode sponge was centered at the onion. Each patient received 30 minutes of real or sham tDCS stimulation in each session, and the sessions were arranged 1 daily for 7 consecutive working days. The trial was administered in a double-blind design meaning that neither the patient nor the physician knew if the patient received the real or sham stimulation.

The primary outcome of the trial was change in the Timed 25-Foot Walk (T25-FW), at which patients were instructed to walk 25 feet as quickly as possible, but safely. We also used following tools for supplementary assessments: the Multiple Sclerosis Walking Scale-12 (MSWS-12), the Fatigue Severity Scale (FSS), the EDSS, and the Modified Ashworth Scale (MAS).^17^ The MSWS-12 is a multi-items rating scale used to assess the perspectives of patients about the impact of MS on their walking ability. It has 12 items and asks the patients to rate how much MS has affected their mobility ability, such as standing, walking, running, and climbing stairs.^18^ We used the Persian-translated version of the MSWS-12 which has excellent psychometric properties.^19^ Fatigue is very common among the patients with MS, affecting two thirds of them,^20^ and FSS is designed for its measurement. We used a Persian-translated version of FSS reported to have very good psychometric properties.^21^ And, EDSS is the most widely used tool for the assessment of patients with MS’ disability. In these supplementary assessment, higher scores mean more disability. 

A physician (H.O.) blinded to the group allocation performed the stimulations and assessments. Outcome assessment was conducted at baseline and after 7 sessions of stimulation.

We used t-test and Fisher’s exact test to compare the real and sham stimulation groups in the baseline characteristics. Then, we applied mixed effect models to study changes of T25-FW in time. The model provides two sets of estimates. The first set included an estimate for the baseline level of T25-FW in the real vs. sham stimulation groups. The second set included an estimate for the slope of change in the T25-FW speed among sham-stimulated group (time) and another estimate for the change in this slope made by the real tDCS stimulation. Next, we repeated the models by inclusion of age, sex, baseline EDSS score, disease duration, and their interaction with time to examine if our findings were confounded by these variables. In further analyses, we repeated our core model but replaced T25-FW with the MSWS-12 and FSS scores. SPSS software (version 22.0, IBM Corporation, Armonk, NY, USA) was used for the analyses.

## Results

We recruited 17 patients with MS, but 4 of them were missed in the follow up, and did not complete the study (2 in the real and 2 in the sham stimulation groups). The 4 patients were not different from the rest in age (P = 0.614), sex (P > 0.999), T25-FW speed (P = 0.572), MSWS-12 score (P = 0.211), and FSS scores (P = 0.921). 

Demographic and baseline clinical characteristics of the 13 patients who completed the trial are presented at table 1. Both relapsing remitting and progressive types of MS were represented in the groups. The real and sham stimulation groups were not different in age, sex, MS type, disease duration, and EDSS score.

**Figure 1:**
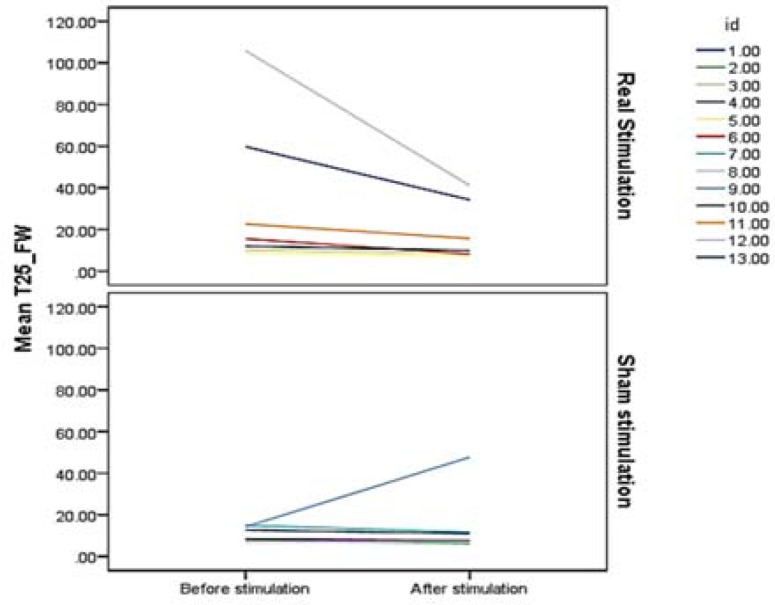
Timed 25-Foot Walk (T25-FW) speed change from before to after transcranial direct current stimulation (tDCS) stimulation

***T25-FW speed*** *:* At the baseline, the two groups were not different in the T25-FW speed. We employed a linear mixed effects model to examine the interaction of tDCS stimulation type with the change of T25-FW speed from before to after the treatment (the core model). On average, patients who received real tDCS stimulation walked faster after 7 sessions of stimulation [Estimate = -2.7, standard error (SE) = 1.3, P = 0.049], while walking speed of sham stimulation recipients did not change (Table 2, Figure 1). Estimates showed that for every session of stimulation, recipients of real tDCS stimulation spent 2.7 seconds less for walking the 25 feet.

In further analyses, we examined if the efficacy of real tDCS stimulation in speeding up walking ability of patients with MS was confounded by patients’ age, sex, baseline EDSS, or disease duration. In successive models, we added these variables and their interaction with time to the core model. However, the result did not change, and real tDCS stimulation decreased the time patients with MS needed to walk 25 feet (Table 2).

**Table 1:** Demographic and clinical characteristics of the patients

**Variable**	**Real stimulation (n = 7)**	**Sham stimulation (n = 6)**	**All**	**P**
	**n (%)**	**n (%)**	**n (%)**	
Women	6 (86)	4 (67)	10 (77)	0.559
MS type				0.559
RRMS	1 (14)	2 (33)	3 (23)	
Progressive	6 (86)	4 (67)	10 (77)	
	**Mean ± SD**	**Mean ± SD**	**Mean ± SD**	
Age (year)	37.9 ± 14.7	40.2 ± 10.1	38.9 ± 12.3	0.751
MS duration (year)	13.9 ± 11.2	9.5 ± 5.1	11.8 ± 8.9	0.401
EDSS	3.7 ± 1.3	3.8 ± 1.1	3.8 ± 1.2	0.867
T25-FW	33.3 ± 36.5	10.8 ± 3.5	23.1 ± 28.5	0.152
MSWS-12	3.6 ± 1.3	3.5 ± 0.8	3.6 ± 1.1	0.851
FSS	4.4 ± 1.7	4.3 ± 1.9	4.3 ± 1.7	0.920

MS: Multiple sclerosis; RRMS: Relapsing remitting multiple sclerosis; SD: Standard deviation; EDSS: Expanded disability status scale; T25-FW: Timed 25-Foot Walk; MSWS-12: Multiple sclerosis walking scale-12; FSS: Fatigue severity scale

**Table 2:** Timed 25-Foot Walk (T25-FW) speed before and after transcranial direct current stimulation (tDCS) stimulation

**Models’ terms**	**Model 1**			**Model 2**		**Model 3**		**Model 4**		**Model 5**	
	**Estimate ** **(SE)**		**P**	**Estimate ** **(SE)**	**P**	**Estimate ** **(SE)**	**P**	**Estimate ** **(SE)**	**P**	**Estimate ** **(SE)**	**P**
Real tDCS	22.9 (11.3)		0.058	24.7 (10.2)	0.025	20.9 (11.3)	0.081	24.4 (6.7)	0.001	27.5 (11.2)	0.024
Time	0.5 (0.9)		0.549	0.4 (2.3)	0.879	0.4 (1.3)	0.792	2.8 (2.1)	0.209	-0.8 (1.1)	0.480
Real tDCS × time	-2.7 (1.3)		0.049	-2.7 (1.3)	0.048	-2.8 (1.3)	0.051	-2.6 (1.2)	0.046	-3.2 (1.1)	0.014
Age				0.8 (0.4)	0.094						
Age × time				0.0 (0.1)	0.932						
Sex						10.5 (13.4)	0.443				
Sex × time						0.3 (1.5)	0.861				
EDSS								16.4 (2.9)	< 0.001		
EDSS × Time								-0.6 (0.5)	0.261		
Disease duration										-1.1 (0.7)	0.116
Disease duration × time										0.1 (0.1)	0.086

SE: Standard error; tDCS: Transcranial direct current stimulation; EDSS: Expanded disability status scale

***Other assessments*** *:* MSWS-12 is a questionnaire which enquires patients with MS to rate the impairment made by their disease in their mobility. At the baseline, the two tDCS groups were not different. We repeated the core model but replaced T25-FW with MSWS-12. The results showed that real tDCS stimulation was not different from sham stimulation in the MSWS-12 scores’ change from before to after the sessions (Real tDCS stimulation × time estimate = -0.0, SE = 0.0, P = 0.275). Then, we repeated the core model and replaced the T25-FW with the FSS score. The results did not show the efficacy of real tDCS stimulation in decreasing fatigue in patients with MS (Real tDCS stimulation × time estimate = -0.1, SE = 0.1, P = 0.089).

Table 3 shows patients’ EDSS scores before and after the treatment. In both groups, EDSS scores did not change after the treatment. Looking at the EDSS functional systems, we did not find any change in the domains’ scores after the treatment except in the bladder function in 2 patients. 

**Table 3:** Expanded disability status scale (EDSS) scores of patients with multiple sclerosis (MS) before and after transcranial direct current stimulation (tDCS) stimulation

**tDCS stimulation**	**EDSS-Pyramidal**		**EDSS-Cerebellar**		**EDSS-Sensory**		**EDSS-Bowel**		**EDSS**	
	**Before**	**After**	**Before**	**After**	**Before**	**After**	**Before**	**After**	**Before**	**After**
Real	3	3	0	0	0	0	2	2	5.0	5.0
Real	3	3	0	0	0	0	2*	1*	3.5	3.5
Real	2	2	0	0	0	0	1	1	2.0	2.0
Real	3	3	1	1	0	0	0	0	3.0	3.0
Real	4	4	0	0	0	0	2*	1*	6.0	6.0
Real	3	3	0	0	0	0	0	0	3.0	3.0
Real	3	3	0	0	1	1	2	2	3.5	3.5
Sham	2	2	0	0	0	0	4	4	4.0	4.0
Sham	3	3	0	0	0	0	2	2	3.5	3.5
Sham	3	3	0	0	1	1	3	3	3.5	3.5
Sham	3	3	0	0	0	0	3	3	6.0	6.0
Sham	3	3	2	2	0	0	0	0	3.0	3.0
Sham	3	3	0	0	0	0	1	1	3.0	3.0

tDCS: Transcranial direct current stimulation; EDSS: Expanded disability status scale

All the patients had score zero at the brainstem, visual, and mental functional systems both before and after the stimulation.

* Patients with MS whose EDSS-Bowel score changed after tDCS stimulation. Other scores did not change.

Two patients were women, 27 and 44 years old, who received real tDCS stimulation, and their bowel and bladder functional system scores improved from 2 to 1 after the treatment. Finally, we compared the groups in the MAS scores. After the treatment, the spasticity MAS score did not change in any of the patients but 1 who received real tDCS stimulation.

## Discussion

The present study showed that tDCS stimulation of the lower limb motor cortex will speed up patients with MS in walking. However, this beneficiary effect was not accompanied by improvement in patients’ mobility in daily activities, reported by the patients. Further studies are needed to replicate this study in a larger sample size, to resolve the discrepancy in the treatment efficacy between the objective and subjective measures of mobility impairments, and to uncover the molecular and cellular mechanisms underlying the faster walking of patients with MS after tDCS stimulation.

MS incidence and prevalence are increasing in Iran.^22^ As life expectancy of patients with MS is more than 70 years,^23^^,^^24^ and about two thirds of them suffer from walking disability after 10 years since diagnosis,^1^^,^^2^ we anticipate of a rise in the number of patients with MS with mobility impairment. So, looking for rehabilitative techniques capable of decreasing difficulty of walking in these patients is a health priority. In this study, we examined the efficacy of tDCS stimulation in ameliorating walking disability of patients with MS due to tDCS efficacy in improving walking ability of patients with stroke^14^ and improving pain,^10^ fatigue,^12^ and tactile sensory deficit^11^ of patients with MS. We found that tDCS improved walking speed in patients with MS, although it was not accompanied by improvement in patients’ walking difficulty in activities of daily living.

To our knowledge, only one other study has investigated use of tDCS stimulation in the treatment of walking difficulty of patients with MS.^25^ The investigators randomized 20 patients with RRMS to either real or sham tDCS stimulation which was implemented for 20 minutes per day for 5 consecutive days. For outcome assessment, they did not use T25-FW test. Instead, they used MAS, MSWS-12, and Multiple Sclerosis Spasticity Scale (MSSS-88), as the study focus was MS-associated spasticity. The study did not show any benefit for tDCS in decreasing lower limb spasticity of patients with MS that is in parallel to our study finding. 

Our study showed the efficacy of tDCS stimulation in increasing walking speed without any effect on the lower limb spasticity. One possible explanation for this finding is the more power we have in using walking speed tests compared to spasticity scales like MAS which has a limited range of change from zero to 4 with floor and ceiling effects. However, it is also possible for other mechanisms to be involved. As a potential mechanism, is tDCS-induced excitation of other brain networks whose activation results in a better walking speed without any effect on decreasing spasticity of the lower limbs. Activation of the brain executive network, including areas like frontal cortex, has been found associated with walking speed,^26^ and this network might be activated in our study. Unfortunately, we did not have any functional imaging to verify this hypothesis. Moreover, by exploiting MSWS-12 questionnaire, we did not find any benefit for the tDCS stimulation in improving mobility in the activities of daily living such as walking, running, and climbing stairs in patients with MS. Two possible logics, at least, can explain this finding, one statistical and one pathophysiological. From the statistical point of view, an objective test like T25-FW, whose score is a continuous ratio variable, is more powerful to detect an intervention effect than a subjective test like MSWS-12, whose score is an interval variable. From the pathophysiological point of view, cerebellar pathways, which are also involved in walking and are frequently compromised in MS, were not stimulated in our study. So, patients with MS had faster walking speed in our T25-FW, but still suffered from walking difficulty because of the gait ataxia they suffered from. We hypothesize that if we had stimulated cerebellum besides the corticospinal pathway, we would have stronger improvement in the walking speed and a net benefit for patients with MS in their activities of daily living. In fact, a recent tDCS study on patients with stroke reported a faster walking when cerebellum was stimulated in addition to the corticospinal system.^27^

Intriguingly, we found incontinence improvement in two patients with MS who received real tDCS stimulation while no improvement occurred in patients with MS under sham stimulation. While we did not have enough sample for a statistical analysis, this finding merits to get addressed in future trials. In fact, overactive bladder and urine incontinence is very common among the patients with MS affecting as much as one third of them.^28^ Although we did not find any study about use of brain stimulation for the treatment of MS-related urine incontinence, sacral and tibial nerve electrical stimulation has been used for the treatment of overactive bladder with modest benefit.^29^

The strength of our study is examining an inexpensive therapy for the treatment of walking disability of patients with MS in a randomized double-blind clinical trial; neither the patient nor the physician was aware of the allocated type of tDCS stimulation. We also utilized both objective and subjective methods to assess therapy efficacy on the walking ability of patients with MS. However, there were some limitations too. The study was performed on only 13 participants; hence, larger trials are necessary. Moreover, we will need functional imaging to unveil brain areas which are activated by tDCS stimulation and mediate its clinical benefit. In addition, two additional arms should be added to future trials; one arm with cerebellar stimulation, and one arm with combined cerebellar and primary motor cortex stimulation. These additional arms will elucidate the best protocols to improve walking disability of patients with MS.

## Conclusion

We found that lower limb motor cortex stimulation using tDCS is beneficial for patients with MS and difficulty in walking; patients walked faster after the stimulation. But, this faster walking was not accompanied by improvement in patients’ reported difficulties in the activities of daily living. To help the patients with MS in suffering less during their daily activities, we should examine addition of other rehabilitation methods to our brain stimulation. In a recent study, investigators found that a combination of aerobic and resistance exercises improved walking ability in patients with MS.^30^ Combination of such exercise therapies with the brain stimulation may synergistically act and improve the walking disability of patients with MS far better than use of one of these interventions.

## Notes:

**How to cite this article:** Oveisgharan Sh, Karimi Z, Abdi S, Sikaroodi H. The use of brain stimulation in the rehabilitation of walking disability in patients with multiple sclerosis: A randomized double-blind clinical trial study. Iran J Neurol 2019; 18(2): 57-63.
